# Ectopic Integration Vectors for Generating Fluorescent Promoter Fusions in *Bacillus subtilis* with Minimal Dark Noise

**DOI:** 10.1371/journal.pone.0098360

**Published:** 2014-05-29

**Authors:** Stephanie Trauth, Ilka B. Bischofs

**Affiliations:** 1 Zentrum für Molekulare Biologie der Universität Heidelberg (ZMBH), Heidelberg, Germany; 2 Center for Quantitative Analysis of Molecular and Cellular Biosystems (BioQuant), University of Heidelberg, Heidelberg, Germany; University of Groningen, Groningen Institute for Biomolecular Sciences and Biotechnology, Netherlands

## Abstract

Fluorescent protein promoter reporters are important tools that are widely used for diverse purposes in microbiology, systems biology and synthetic biology and considerable engineering efforts are still geared at improving the sensitivity of the reporter systems. Here we focus on dark noise, i.e. the signal that is generated by the empty vector control. We quantitatively characterize the dark noise of a few common bacterial reporter systems by single cell microscopy. All benchmarked reporter systems generated significant amounts of dark noise that exceed the cellular autofluorescence to different extents. We then reengineered a multicolor set of fluorescent ectopic integration vectors for *Bacillus subtilis* by introducing a terminator immediately upstream of the promoter insertion site, resulting in an up to 2.7-fold reduction of noise levels. The sensitivity and dynamic range of the new high-performance pXFP_Star reporter system is only limited by cellular autofluorescence. Moreover, based on studies of the *rapE* promoter of *B. subtilis* we show that the new pXFP_Star reporter system reliably reports on the weak activity of the *rapE* promoter whereas the original reporter system fails because of transcriptional interference. Since the pXFP_Star reporter system properly isolates the promoter from spurious transcripts, it is a particularly suitable tool for quantitative characterization of weak promoters in *B. subtilis*.

## Introduction

Fusions between promoters and fluorescent reporter genes have emerged as important study tools that serve many purposes in molecular and applied microbiology, systems biology and synthetic biology. When the promoter is activated bacteria produce a fluorescent protein and - depending on the properties of the fluorescent protein - fluorescence can typically be observed within minutes. The application spectrum of fluorescent gene reporters ranges from the identification and molecular characterization of *cis* acting elements, the monitoring of gene expression dynamics in real-time in bacterial bulk populations [1] or individual cells [2], the assessment of population heterogeneity in gene expression, cell phenotype mapping in bacterial micro-colonies and biofilms [3], [4] to diverse applications of fluorescent protein promoter fusions in biosensors [5].

The engineering and benchmarking of tools to facilitate studies with fluorescent promoter fusions is still an area of active research [6]–[9]. For any reporter assay its sensitivity is an essential factor. In general, the sensitivity is defined by the signal-to-noise ratio of the read-out signal. For promoter reporters, the immediate signal is the mRNA that is generated under the control of the promoter of interest. The biological read-out of fluorescent promoter fusions is the amount of fluorescent protein that is being produced from the mRNA. Upon excitation the fluorescent protein variant emits a characteristic number of photons to yield a physical signal that is finally converted into the electronic read-out by the detector. Two complementary strategies can be used to improve the signal-to-noise ratio: firstly, to specifically amplify the signal before the final read-out or secondly, to decrease the noise. Both strategies can be applied on all three levels concerning the primary biological, the secondary biophysical and the tertiary electronic read-out signal by engineering the properties of the vector (vector engineering), the fluorescent protein (protein engineering) or the detector (instrument engineering) respectively. Currently used detectors of fluorescence are (microplate) photometers, flow cytometers and microscopes. In particular, microscopy systems nowadays offer remarkable sensitivity allowing for highly quantitative measurements with molecular resolution even with conventional epi-fluorescence microscopy [10]. Many fluorescent protein variants have been developed and their properties have been continuously improved by protein engineering [11], [12]. In this work we focus on optimizing the signal-to-noise ratio on the level of the primary biological signal by engineering the properties of the vector. Compared to enzymatic assays, fluorescence assays are generally less sensitive. When an enzyme, e.g. β-galactosidase, is expressed, the generated protein signal is further amplified by the reaction that is catalyzed by the enzyme, which produces the final read-out signal. Fluorescent reporters lack this intrinsic amplification potential. Hence, fluorescent reporter engineering has focused on optimizing fluorescent protein expression in order to amplify the signal e.g. by using optimal ribosome-binding sites, optimizing codon usage, boosting translation by enhancer sequences or by having several fluorescent proteins being transcribed in an operon [6], [9], [13]–[15]. On the other hand, noise engineering has received less attention.

The read-out produced by a (photo)-detector in the absence of a signal is generally referred as “dark noise”. In analogy, one may define the read-out produced by a biological reporter system in the absence of the signal (i.e. the promoter of interest) as dark noise. For fluorescent reporters one can distinguish between two kinds of dark noise: general dark noise and specific dark noise. General dark noise is defined as the amount of noise produced by the cell in the untransformed state, i.e. the cellular autofluorescence. Specific dark noise is the additional amount of noise produced by the “empty” reporter, i.e. the reporter without an inserted target promoter (but with a translation signal present). Theoretically, the mere introduction of a reporter into the cell could influence the cellular physiology and thereby change the autofluorescence properties of the cell. In addition, specific dark noise could originate from spurious transcripts that are initiated from other loci than the promoter of interest. This could result in the expression of the reporter protein even in the absence of the promoter of interest and hence contribute to noise. Gene expression resulting from “empty” constructs is a well-known phenomenon that has been mainly observed in enzymatic reporter systems for vertebrate cell lines, where it is attributed to transcription initiation from cryptic promoter elements or regulatory elements present in the vector backbone [16], [17]. Noise originating from spurious transcripts will be amplified to a similar extent as the actual signal generated by the promoter of interest. Due to the large degree of signal amplification in enzymatic reporter systems, specific dark noise has been a common problem in such reporter systems in the past [16], [18] and in many cases it was the dominant contribution to the total noise. In contrast for fluorescence reporters, the general noise is expected to dominate as cells generate significant amount of autofluorescence and because spurious transcription noise is amplified to a lesser extent by the reporter system. This might be why for fluorescent reporter systems specific noise received little attention so far. However, as the sensitivity of fluorescent reporter systems and detectors are continuously improving, specific dark noise might become an important limitation of fluorescent reporter systems. Moreover, it is well-known that two transcriptional processes can directly influence each other [19], [20]. Thus the promoter-based transcription and the spurious transcription process could interfere with each other in unpredictable ways. Such interference could therefore render reporter systems suffering from specific noise susceptible to generating artefacts and may preclude measurements of promoter activities.

Here we introduce benchmarking characteristics in order to quantify the dark noise in a few current reporter systems for *Bacillus subtilis* and *Escherichia coli* [1], [9]. All strains generated considerable amounts of dark noise that exceeded the cellular autofluorescence. By reengineering a multi-color set of fluorescent integration vectors for *B. subtilis* we obtained an up to 2.7-fold improvement of the noise level with respect to the parent reporters. We also demonstrate that the presence of specific dark noise in the parental reporter system compromises the measurement of weak promoter activities and show that proper buffering of spurious transcripts by a transcriptional terminator in the new reporter system is necessary for being able to infer the activity of the *rapE* promoter of *Bacillus subtilis*.

## Methods

### Bacterial strains, media and growth conditions

*Escherichia coli* DH5α (Invitrogen, Carlsbad, CA, USA) was used for routine cloning. *Bacillus subtilis* 168 1A700 strain was used as parental strain. A list of all strains is given in Table 1. Strains were grown on low salt Lysogeny Broth (LB) (10 g/L Bacto tryptone, 5 g/L NaCl, 5 g/L Bacto yeast extract) at 37°C with 180 rpm shaking or solidified with 1.5% (w/v) Bacto agar. Appropriate antibiotics were supplemented to the medium when required: 100 µg/mL ampicillin, 50 µg/mL kanamycin for *Escherichia coli* and 5 µg/mL chloramphenicol, 10 µg/mL kanamycin for *Bacillus subtilis*.

**Table 1:** Strains and plasmids.

Strains/Plasmids	Genotype	Source
**Plasmids**		
**pGFPamy**	‘*amyE cat* LIC *promoterless gfpmut3 amyE*’ *bla* ColE1 origin	[9]
**pYFPamy**	‘*amyE cat* LIC *promoterless iyfp amyE*’ *bla* ColE1 origin	[9]
**pCFPamy**	‘*amyE cat* LIC *promoterless cfp_Bs_ amyE*’ *bla* ColE1 origin	[9]
**pGFPbglS**	*bglS′* LIC *promoterless gfpmut3 nptIII ′bglS bla* ColE1 origin f1(+)origin	[9]
**pGFP_Star**	‘*amyE cat* T*gyrA* LIC *promoterless gfpmut3 amyE*’ *bla* ColE1 origin	This study
**pYFP_Star**	‘*amyE cat* T*gyrA* LIC *promoterless iyfp amyE*’ *bla* ColE1 origin	This study
**pCFP_Star**	‘*amyE cat* T*gyrA* LIC *promoterless cfp_Bs_ amyE*’ *bla* ColE1 origin	This study
**pGFP_Star_P** ***rapE***	‘*amyE cat* T*gyrA* P*rapE*-*gfpmut3 amyE*’ *bla* ColE1 origin	This study
**pGFPamy_P** ***rapE***	‘*amyE cat* P*rapE*-*gfpmut3 amyE*’ *bla* ColE1 origin	This study
**Strains**		
***E. coli***		
**DH5α**	F^−^ φ80*lac*ZΔM15 Δ(*lac*ZYA-*arg*F)U169 *rec*A1 *end*A1 *hsd*R17(rk^−^, mk^+^) *pho*A *sup*E44 *thi*-1 *gyr*A96 *rel*A1 λ^−^	Invitrogen
**BW25113**	F^−^ *Δ(araD-araB)567 ΔlacZ4787*(::*rrnB*-3) *λ^−^ rph-1 Δ(rhaD-rhaB)568 hsdR514*	[35]; Gift of Karl Kochanowski
**BW25113_pUA139**	F^−^ *Δ(araD-araB)567 ΔlacZ4787*(::*rrnB*-3) *λ^−^ rph-1 Δ(rhaD-rhaB)568 hsdR514 nptII promoterless gfpmut2* sc101 origin	Strain [35]; Plasmid [1]; Gift of Karl Kochanowski
***B. subtilis***		
**168 1A700**	*trpC2*	Gift of Oscar Kuipers
**168_pGFPamy** *	*trpC2 amyE:: promoterless gfpmut3 cat*	This study
**168_pYFPamy** *	*trpC2 amyE:: promoterless iyfp cat*	This study
**168_pCFPamy** *	*trpC2 amyE:: promoterless cfp_Bs_ cat*	This study
**168_pGFP_Star** *	*trpC2 amyE::* T*gyrA promoterless gfpmut3 cat*	This study
**168_pYFP_Star** *	*trpC2 amyE::* T*gyrA promoterless iyfp cat*	This study
**168_pCFP_Star** *	*trpC2 amyE::* T*gyrA promoterless cfp_Bs_ cat*	This study
**168_pGFPbglS**	*trpC2 bglS:: promoterless gfpmut3 nptIII*	This study
**168_P** ***rapE-gfp*** ** (Star)** *	*trpC2 amyE::* T*gyrA* P*rapE-gfpmut3 cat*	This study
**168_P** ***rapE-gfp*** ** (amy)** *	*trpC2 amyE::* P*rapE-gfpmut3 cat*	This study

* two independent clones are used for this study.

### Recombinant DNA techniques and oligonucleotides

Oligonucleotides (Sigma-Aldrich, Steinheim, Germany) used in this study are listed in Table S1. Kits and enzymes given in Table S2 were used according to the user manuals. DNA recombination was performed according to standard procedures [21].

### Plasmid construction

All plasmids used in this study are listed in Table 1. For the construction of pGFP_Star, the terminator of the *B. subtilis gyrA* gene (T*gyrA*) was cloned upstream of the ligation-independent cloning (LIC) site of pGFPamy [9]. T*gyrA* was amplified with the primers ST135 and ST147 from genomic DNA of *B. subtilis* 168 1A700. The PCR product and pGFPamy were subsequently cleaved with SacII, ligated and transformed into *E. coli* resulting in plasmid pre-pGFP_Star. Integration and orientation of T*gyrA* was checked by PCR. Constructs were sequenced using primers ST16 and ST17.

The YFP and CFP vector variants were constructed by Gibson assembly [22]. A portion of the vector pre-pGFP_Star that included T*gyrA* was amplified using primers ST163 and ST162. The fluorophore containing parts of pYFPamy or pCFPamy were amplified using primers ST161 and ST164. Both parts were joined by Gibson assembly. Clones were checked by colony PCR using the ST17 primer and primers LA20 (*cfp*) and LA21 (*iyfp*), respectively. Correct assembly of DNA was verified by analytical restriction digest with the enzyme combinations SalI/NdeI and SalI/ScaI. The plasmid regions containing the inserted terminator and LIC site were sequenced with the primers used for colony PCR.

Finally, *ldh* homologous parts present in the pXFPamy derived pre-pXFP_Star plasmids, which lead to a 228 bp deletion in the *ldh* gene locus in some *Bacillus* transformants, were deleted using Gibson assembly. The pXFP_Star plasmids were amplified with primers ST189 and ST190 using pre-pXFP_Star as templates, which lead to PCR fragments lacking the homologous *ldh* parts (96 bp), and then re-circularized by Gibson assembly. Candidates were identified by colony PCR using primers ST191 and ST192. Correct assembly of DNA was verified by analytical restriction digest with the enzyme combinations SalI/NdeI and SalI/ScaI. The final pXFP_Star plasmids - pGFP_Star (7380 bp), pYFP_Star (7410 bp), pCFP_Star (7255 bp) - were sequenced with primers ST191, ST192, ST41 and ST17, as well as the fluorophore specific primers ST16 (*gfpmut3*), ST193 (*iyfp*) and ST194 (*cfp_Bs_*) respectively.

For the *B. subtilis rapE* promoter reporter fusions, a *rapE* promoter construct was amplified that includes the putative ComA and the CodY binding sites [23]. The CodY binding site is located within the *rapE* coding sequence [23] (nucleotide position 224–242 downstream of the start codon **T**TG, with **T** defined as +1). To avoid translation of undesirable fusion proteins under promoter activation or a putative expression of fragmented proteins in the cell under CodY repression, the construct was specially designed to lack the ribosome binding site and the start codon. This is reflected in the cloning procedure. Upstream and downstream parts of the *B. subtilis rapE* promoter were amplified from genomic DNA of *B. subtilis* 168 1A700 using primers ST165 and ST166 (upstream fragment) or ST169 and ST168 (downstream fragment). The upstream and downstream parts were fused by overlap extension PCR. The plasmids pGFPamy and pGFP_Star were cleaved with SmaI prior to T4 DNA polymerase treatment. For the LIC procedure, 40 fmol of the vectors were treated with 0.6 U of T4 DNA polymerase in presence of 2.5 mM dATP and 200 fmol of the promoter construct (708 bp) were treated with 0.6 U of T4 DNA polymerase in presence of 2.5 mM dTTP. Samples were incubated for 20 min at 22°C and inactivated for 30 min at 75°C. After T4 DNA polymerase treatment, 5 fmol of the vector were annealed with the insert in a molar ratio 1∶5 for 10 min at room temperature and transformed into *E.coli*. Transformants were checked by colony PCR and sequenced using primers ST16 and ST17.

### *Bacillus subtilis* strain construction

*B. subtilis* was transformed using a standard method [24]. Transformants were screened for construct integration into the *amyE* locus by lack of amylase activity on LB plates containing 1% (w/v) starch followed by treatment with Lugol's solution. Clones were verified by sequencing PCR products obtained from genomic DNA with primers ST39 and ST40. *ldh* deletion strains were discarded. Finally, the absence of single cross-over events was checked by PCR with primers ST129 and ST130 on genomic DNA. For *Bacillus* strains containing *amyE* integrated reporters two independent clones were stored and used in this study. For *bglS* locus integration cells were transformed with BamHI linearized DNA and checked by colony PCR with primers ST16 and LA38.

### Benchmarking assay

Strains were inoculated from an LB overnight culture into a 20 mL LB culture without antibiotics with a starting optical density at 600 nm (OD_600nm_) of 0.02. Strains were cultivated to an OD_600nm_ of 0.3, 3 and 5 (*B. subtilis*) or an OD_600nm_ of 4 (*E. coli*) at 37°C and 180 rpm shaking. Cells were harvested and washed once in M9 medium [25] supplemented with 10 µg/mL erythromycin for *B. subtilis* and 150 µg/mL for *E. coli* to stop protein expression. Samples were resuspended to a final OD_600nm_ of 2 and 2 µl were spread on a gel pad made of the same medium solidified with 1.5% (w/v) Ultra Pure Agarose (Invitrogen). Samples were sealed with Baysilone high viscose silicone paste (Bayer, Leverkusen, Germany) and covered by a coverslip.

Brightfield and fluorescence images were taken on a DeltaVision Elite Imaging System (Applied Precision, Issaquah, WA, USA) equipped with Olympus IX71 microscope, a solid state illumination unit and a CoolSNAP HQ2 camera. Samples were imaged with an UPlanSApo 100×/1.40na oil objective (Olympus, Tokyo, Japan) with the following exposure settings and filter sets (excitation, emission): GFP: 0.2 s with 100% excitation (475 nm/28, 525 nm/50), YFP: 1 s exposure with 100% excitation (513 nm/17, 559 nm/38) and CFP: 2 s exposure with 100% excitation (438 nm/24, 470 nm/24). Images were binned 2×2 using SoftWoRx 5.5 software.

Brightfield images were segmented using a customized program QFTrack [26] written in MatlabR2011b. Segmentation performance was manually inspected. For each cell the mean fluorescence intensity was determined from the segmented area and the background fluorescence subtracted. For each condition 2500–7000 cells were analyzed. All strains were at least measured twice including two independent clones (except for *E.coli* and *B. subtilis* 168_pGFPbglS).

### Promoter assay

The promoter assays were performed according to the benchmarking protocol with the following modifications: Cells were cultivated to an OD_600nm_ of 2.5 and 6. Samples were imaged with a PCO Edge sCMOS camera with the following exposure settings: GFP: 0.08 s with 100% excitation.

### Plasmid and sequence accession

Plasmids have been made available through the Bacillus Genetic Stock Center (Columbus, OH, USA) with the following accession numbers: pGFP_Star: ECE295, pYFP_Star: ECE296, pCFP_Star: ECE297. Complete vector sequences are accessible at NCBI GenBank with accession numbers: pGFP_Star: KJ411636, pYFP_Star: KJ411637, pCFP_Star: KJ411638.

## Results and Discussion

### Benchmarking dark noise in current bacterial fluorescent reporter systems

**Figure 1:**
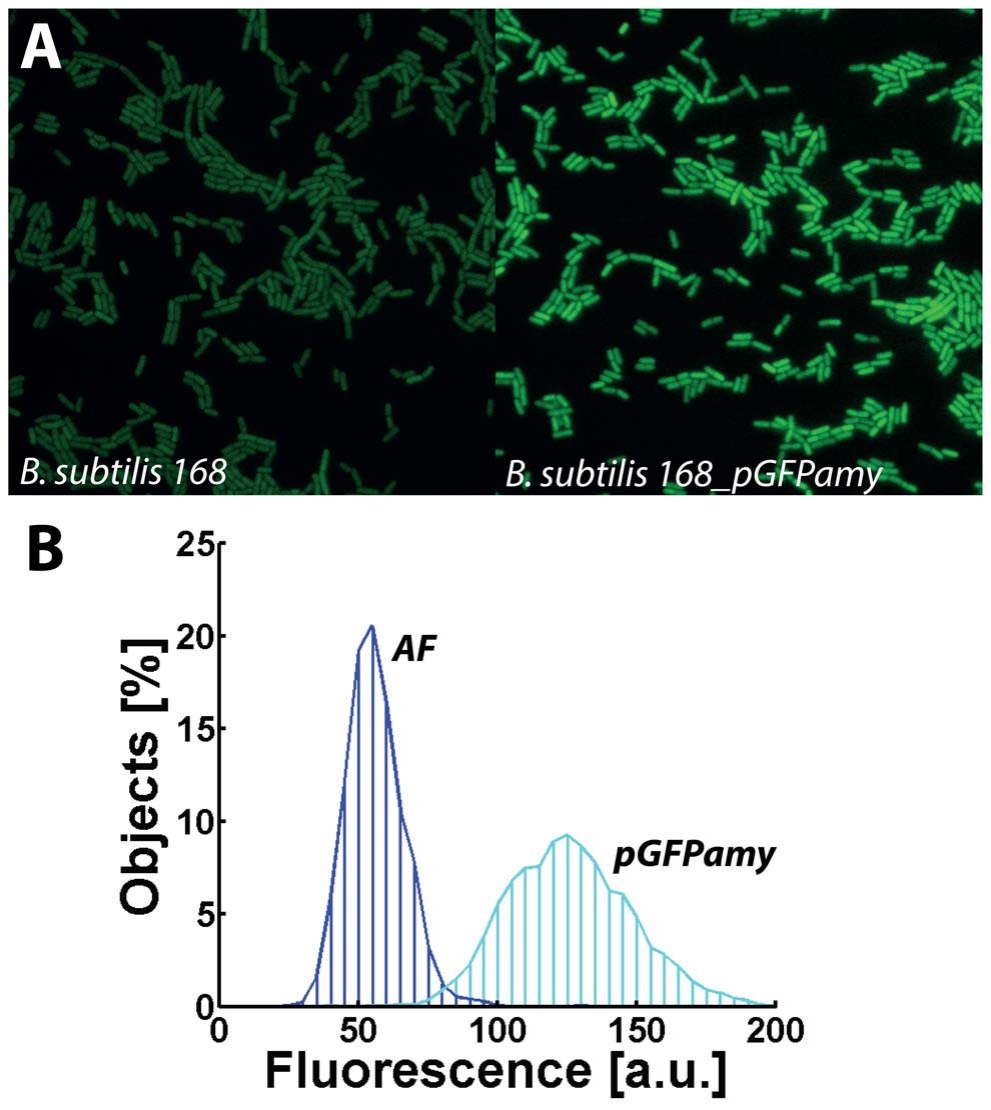
Dark noise generated by pGFPamy. *B. subtilis* 168 transformed with the empty vector pGFPamy causes elevated fluorescence compared to the cellular autofluorescence (AF) of the parental strain: A) Fluorescence microscopy snapshots and B) fluorescence intensity distributions at OD_600nm_ of 5.

To quantitatively characterize different reporter systems we measured the fluorescence intensity of individual cells transformed with the respective “empty vector” by fluorescence microscopy and compared it to cellular autofluorescence in untransformed cells. We tested all color variants (*gfpmut3*, *iyfp cfp_Bs_*) from the pXFPamy family of fluorescent *amyE* integration vectors in *Bacillus subtilis* [9], the pGFPbglS vector [9] and the empty plasmid of the GFP-promoter library developed for *E. coli* [1]. Figure 1 shows the histogram of the fluorescence intensities for pGFPamy. In comparison to the autofluorescence generated by cells of the parental strain, the transformed cells generate on average about two-fold more fluorescence and the cell-to-cell variability of fluorescence is also twice as large as indicated by the broader intensity distribution.

**Figure 2:**
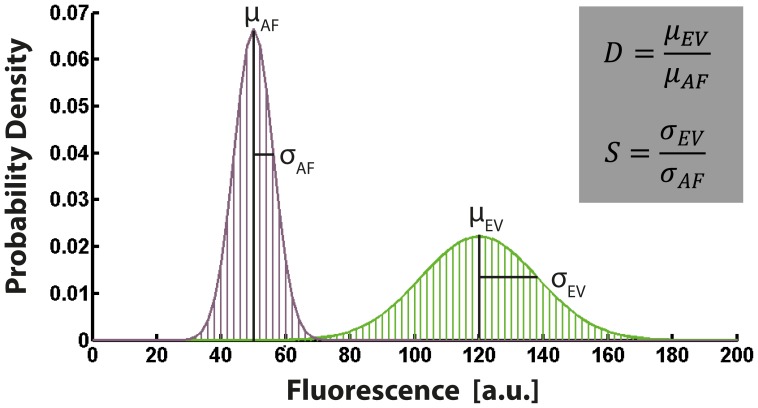
Dark noise benchmarking characteristics. The benchmarking parameters D and S are defined as the ratio of the mean intensities (μ) and the standard deviations (σ) of the respective intensity distributions originating from the empty vector control (EV) and the autofluorescence (AF) of the parental strain. D is correlated to the accessible dynamic range and S is correlated to the sensitivity of the reporter system.

To quantitatively benchmark the performance of different vector systems we introduce two parameters related to the accessible dynamic range and sensitivity of a given reporter system, as shown in Figure 2. We define 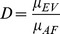 as the ratio of the mean intensities (μ) of the respective intensity distributions originating from the autofluorescence (AF) and the empty vector (EV) control. The larger D, the smaller will be the dynamic range that is accessible with the given reporter system. We also define 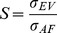 as the ratio of the standard deviation (σ) of the two respective intensity distributions originating from the autofluorescence and the empty vector. With increasing S, the sensitivity of the reporter system will decrease. By introducing D and S we characterize the total dark noise generated by a vector with respect to the general noise. For “ideal” reporter systems, i.e. D = 1 and S = 1 implying optimal vector performance, there is negligible specific dark noise and the reporter system is limited entirely by the autofluorescence.

Table 2 summarizes our benchmarking results for the tested reporter systems. D-values range from 1.16 to 2.13 and S from 1.21 to 2.69. Moreover, the performance characteristics are dependent both on the vector backbone (e.g. pGFPamy and pGFPbglS both drive the same fluorophore *gfpmut3* in *B. subtilis*, but pGFPbglS has better noise properties), the fluorophore (e.g. different fluorescence variants of pXFPamy show different performances), and the host (e.g. the shuttle vector pGFPbglS generates a considerable stronger signal than pGFPamy in *E. coli* (data not shown)). Furthermore, for pGFPamy *D* and *S* also change during growth (Table 3).

**Table 2:** 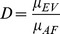
 defined as the ratio of mean fluorescence (μ) and 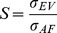 defined as the ratio of the standard deviation (σ) of the fluorescence distribution of empty vector (EV) control and autofluorescence (AF) that were obtained for common *B. subtilis* and *E. coli* promoter reporter fusion vectors.

		D	S
**pGFPamy**	**(** ***B. subtilis*** **)** *	2.13±0.20	2.15±0.39
**pYFPamy**	**(** ***B. subtilis*** **)** *	1.87±0.12	2.69±0.18
**pCFPamy**	**(** ***B. subtilis*** **)** *	1.16±0.08	1.21±0.09
**pGFPbglS**	**(** ***B. subtilis*** **)** *	1.47±0.07	1.46±0.13
**pUA139**	**(** ***E. coli*** **)** #	1.22	1.25

* OD_600nm_ = 5;

# OD_600nm_ = 4.

**Table 3:** Time-dependence in D and S characteristics for pGFPamy and pGFP_Star.

	pGFPamy		pGFP_Star	
	D	S	D	S
**OD_600nm_ = 0.3**	1.25±0.11	1.31±0.20	1.01±0.05	1.05±0.05
**OD_600nm_ = 3**	1.51±0.06	1.37±0.04	1.02±0.03	0.97±0.05
**OD_600nm_ = 5**	2.13±0.20	2.15±0.39	1.07±0.10	0.97±0.14

Thus, all tested vector systems behave sub-optimal with respect to their noise properties. This is reminiscent of the noise observed for enzymatic reporters. It seems likely that specific noise results from upstream transcription. To improve the function of enzymatic vector systems suffering from specific dark noise, there have been several reporter engineering strategies applied in the past: first, to remove the sequence elements responsible for the initiation of transcription or second to terminate the spurious transcripts [16], [18], [27]. The first approach requires *a priori* knowledge about the putative sequence element causing spurious transcription, which is generally not available. A more general and straight-forward strategy is therefore to reduce the specific dark noise by appropriately terminating the respective transcripts. We thus analyzed the sequence of the tested vectors to check whether a terminator had been included in the vector design. It appears that all tested vectors lack a terminator, suggesting that there is room for further improvement.

### pXFP_Star - a high-performance set of multi-color *amyE* integration vectors for *B. subtilis*

**Figure 3:**
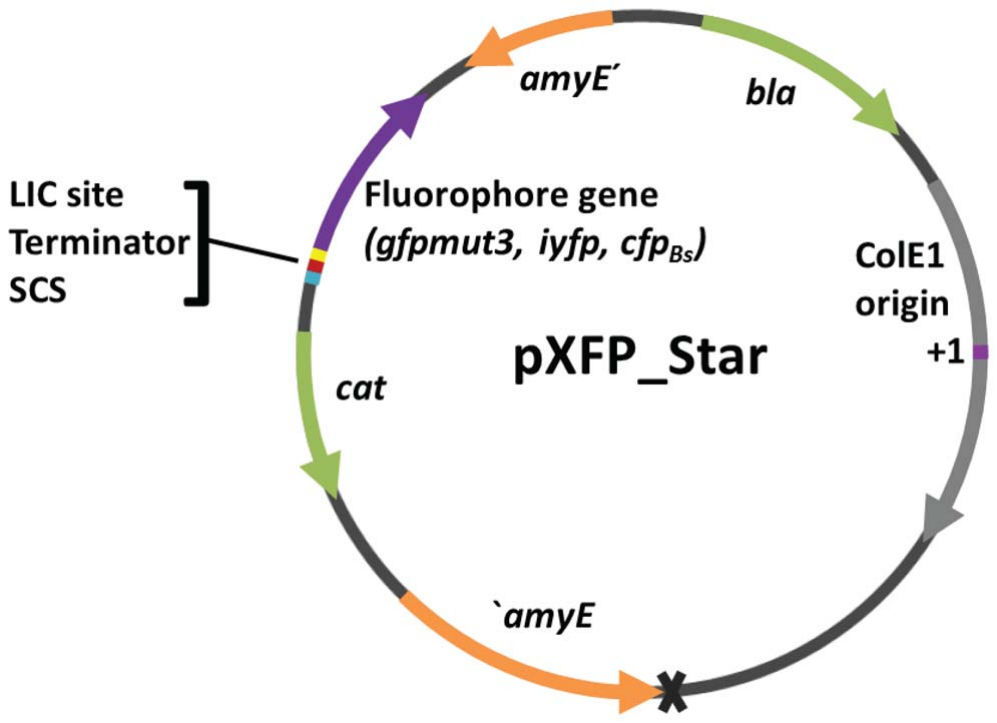
Map of the high-performance *amyE* integration vectors pXFP_Star. The pXFP_Star vector set features the transcriptional terminator of the *B. subtilis gyrA* gene and a small cloning site (SCS) upstream of the LIC site used for ligation-independent cloning of promoter inserts. The grey cross marks the position of the deleted *ldh* parts. The vector variants differ only with respect to the fluorophore *gfpmut3*, *iyfp* or *cfp_Bs_*. The other elements, annotated as *amyE*′ =  *amyE* front, ′*amyE* =  *amyE* back, denote homologous *amyE* parts enabling chromosomal integration into the *amyE* locus. β*-lactamase* gene (*bla*) and *chloramphenicol acetyltransferase* gene (*cat*) are required for selection in *E. coli* and *B. subtilis* respectively. The grey arrow denotes the ColE1 region required for plasmid replication with the ColE1 origin of DNA replication (+1) highlighted in magenta.

For quantitative promoter studies chromosomally integrated reporter fusions are preferred. The *amyE* locus in *Bacillus subtilis* is a common and convenient neutral integration site. We therefore chose the pXFPamy vectors [9] to test whether insertion of a terminator would improve the noise properties. We chose the rho-independent transcription terminator of the essential *Bacillus subtilis DNA gyrase subunit A* gene (T*gyrA*). This terminator perfectly matches the average *Bacillus* terminator in terms of length of the stem as well as the loop of the stem-loop structure, length and sequence of the T stretch and Gibbs free energy [28]. Furthermore, the sequence of the stem does not include mismatches. We cloned T*gyrA* directly upstream of the LIC site, more precisely in the SacII site within the LIC site to avoid that residual parts of a putative promoter remain present downstream of the terminator. We restored the LIC site by joining the restoring parts of the LIC site downstream of T*gyrA* and introduced a small cloning site (SCS) including KpnI and BamHI restriction sites upstream of the terminator to facilitate integration of additional elements, such as a second color or an expression cassette. We then reengineered the other fluorophore vectors by Gibson assembly [22]. While sequencing *Bacillus* cells transformed with pXFPamy or the pre-XFP_Star plasmids, we noticed that some clones had a 228 bp deletion in the *ldh* gene locus, which includes the ribosome binding site and extends into the coding sequence. This deletion is probably the result of a recombination event between a small portion of the *ldh* gene locus present in the pXFPamy vectors [9] and the chromosomal gene locus. *ldh* deletion mutants also show a brighter and more transparent phenotype on LB plates after several days of cultivation (data not shown). Therefore one should check carefully for deletion mutants. In order to optimize the *amyE* integration properties we removed the *ldh* sequence using Gibson assembly. This resulted in the final new multi-color set of integration vectors named pXFP_Star that is suitable for high-throughput applications (Figure 3).

**Figure 4:**
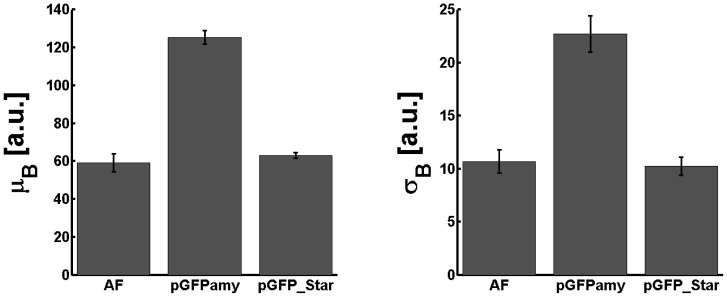
Mean fluorescence and standard deviation of pGFP_Star are reduced to autofluorescence level. Average mean fluorescence (left) and average standard deviation (right) obtained from the fluorescence distributions of the autofluorescence (AF) of *B. subtilis* 168 parental strain and cells transformed with pGFPamy or pGFP_Star “empty” vectors at OD_600nm_ of 5. Results include data of four independent experiments involving two separate clones. Error bars indicate the standard error of the mean.

The new vectors were benchmarked according to the procedure described above by measuring the fluorescence distribution from four independent experiments involving two separate clones. Figure 4 shows the average mean fluorescence and the average standard deviation obtained from the fluorescence distributions for pGFPamy, pGFP_Star and the autofluorescence distributions. The mean fluorescence and the standard deviation in pGFP_Star populations are strongly reduced. Moreover, the statistical footprint obtained from the new pGFP_Star vector matches the respective cellular autofluorescence. Table 3 indicates that for pGFP_Star the D and S values stayed close to 1 over the entire growth phases. Similar results were obtained for the other pXFP_Star vectors, which had both also strongly reduced specific dark noise (Table 4).

**Table 4:** D and S characteristics for pXFP_Star family at OD_600nm_ of 5.

	D	S
**pGFP_Star**	1.07±0.10	0.97±0.14
**pYFP_Star**	1.05±0.06	1.10±0.07
**pCFP_Star**	0.87±0.06	0.98±0.07

Taken together, these results suggest that the performance of the original constructs is indeed hampered by spurious transcription which results in significant expression of fluorescent proteins in the absence of the target promoter. Spurious transcription and imperfect transcription termination are common in *Bacillus subtilis* [29]. Apparently the inserted T*gyrA* works very efficiently to block the spurious transcription so that few if any fluorescent proteins are being expressed in the empty vector control resulting in optimal noise performance. Moreover, terminator function was apparently also maintained throughout growth. In comparison to the parent vector the new high performance set achieves an up to 2.7-fold improvement of the noise, which will improve the signal-to-noise ratio and increase the sensitivity of the reporters accordingly. The varying amounts of relative improvement for the different variants of the pXFP_Star vectors compared to the parent vectors are probably caused by the different amounts of cellular autofluorescence generated on each wavelength and the different brightness of each fluorophore. The pXFP_Star vector set thus has the desired low noise properties (at least under the tested conditions) making it an ideal tool for quantitative promoter studies in *Bacillus subtilis*.

### pXFP_Star plasmids prevent transcriptional interference and enable reliable measurements of weak promoter activities

**Figure 5:**
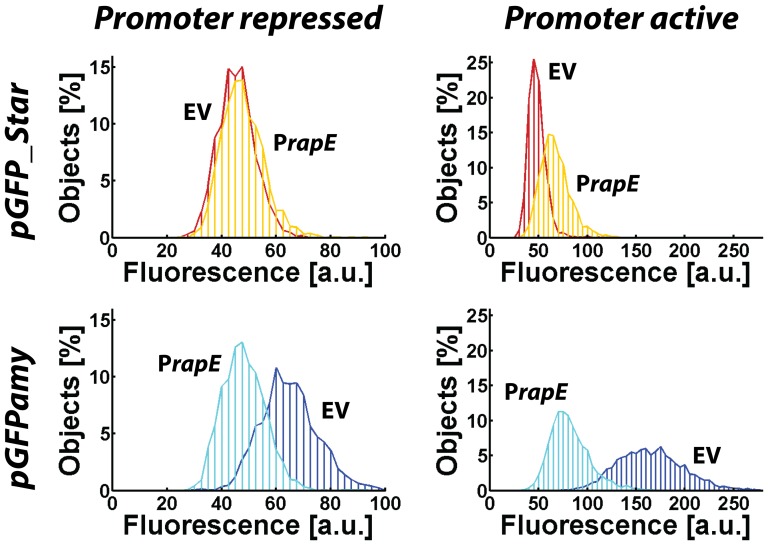
Analysis of *rapE* promoter fusions with the pGFP_Star and pGFPamy reporter system. Histograms of the fluorescence intensity distribution for the *rapE* promoter fusion (P*rapE*) and the “empty” vector reference strain (EV) obtained with the pGFP_Star (top panel) and the pGFPamy (bottom panel) reporter system, respectively. The left column shows the results measured at OD_600nm_ = 2.5 when the *rapE* promoter is repressed and the right panel shows results obtained at OD_600nm_ = 6 when the promoter is weakly active. For the pGFPamy reporter system *PrapE* cells produce less fluorescence than the EV. Hence, spurious upstream transcription and *rapE* promoter transcription do not contribute additively to the fluorescence signal but interfere with each other in unpredictable manners. The Star-system is capable to reliably report on *rapE* promoter activity.

To quantitatively investigate how the presence or absence of specific dark noise affects the measurement of actual promoter activities we focused on the *B. subtilis rapE* promoter. RapE is a phosphatase which acts as inhibitor of the sporulation phosphorelay [30]. Its transcription is repressed by CodY [23], [31] and activated by ComA [30]. During exponential growth in LB transcription is tightly repressed while in stationary phase *rapE* is weakly transcribed [29], [32]. We measured the fluorescence of promoter fusions in exponential and stationary phase with the pGFPamy and pGFP_Star reporter systems. Figure 5 shows the fluorescence distributions obtained for cells expressing the promoter fusions in relationship to the appropriate reference strains carrying the “empty” vector parts. Measurements of the promoter activity of *rapE* using the Star-reporter system (top row) show the expected behavior. In exponential phase (left panel) the fluorescence distribution of the *rapE* promoter fusion (orange line) coincides with the fluorescence distribution of the “empty” vector control (red line), indicating that the promoter is tightly repressed and GFP is not expressed. In stationary phase (right panel) the fluorescence distributions of the *rapE* promoter fusion is shifted to higher fluorescence values, indicating that the *rapE* promoter is active and GFP is expressed. However, when we conduct the same measurement with the parental reporter system pGFPamy (bottom row), we observe that the fluorescence distribution of the *rapE* promoter fusion (cyan line) is shifted to lower fluorescence intensity values compared to the reference strain (blue line) both in exponential and in stationary phase. Apparently cells carrying the *rapE* promoter fusion produce less GFP than cells carrying the “empty” vector control. Therefore the contributions to the fluorescence signal originating from the spurious upstream transcription and transcription from the *rapE* promoter do not add as one might naively expect. Instead the two transcriptional processes influence each other in more complex ways providing a clear indication of transcriptional interference [19], [20]. Note, that the measured intensity distribution in stationary phase when the *rapE* promoter is active is broader when measured with the parental reporter system compared to the Star-system. We therefore conclude that is not possible to quantitatively infer on the promoter activity of *rapE* using the parental reporter system as the transcriptional processes influence each other in unpredictable ways. This case study therefore strikingly demonstrates how transcriptional interference severely compromises studies of weak promoters with reporter systems that are not properly buffered against specific dark noise.

Quantitative promoter assays gain increasing importance for studies in systems and synthetic biology. Measuring signals from weak promoters can be challenging. Especially microscopy-based assays are very promising to accurately quantify the weak signals by deconvolution [10], [33], [34]. To this end, the intensity distribution measured in the promoter fusion reporter strain must be deconvolved with the total dark noise of the empty vector control. It is important to remember that such deconvolution approaches require that the dark noise and the actual signal from the promoter do not influence each other and both contributions will superimpose linearly. This assumption holds for the general dark noise due to cellular autofluorescence but it does not necessarily hold for specific dark noise when transcriptional interference is present.

To conclude, our data suggests that current bacterial fluorescent reporter systems lacking transcriptional terminators may suffer from spurious transcripts, which can severely compromise their ability to faithfully report on the activity of weak promoters. This reinforces the general notion of terminators as important vector design elements. Transcriptional terminators were successfully integrated in the new high-performance multi-color vector suite pXFP_Star to enable a sensitive, robust and accurate quantification of weak promoter activities in *B. subtilis*.

## Supporting Information

Table S1 Oligonucleotides.
(DOCX)

Table S2 Enzymes and kits.
(DOCX)
