# Supplementary material for: Ectopic Integration Vectors for Generating Fluorescent Promoter Fusions in Bacillus subtilis with Minimal Dark Noise
Source: PLoS One. 2014 May 29;9(5):e98360. doi: 10.1371/journal.pone.0098360 (PMC4038550; doi:10.1371/journal.pone.0098360)
Supplement: Table S1 — Oligonucleotides. (DOCX) [file pone.0098360.s001.docx]

**Supporting Information**

**Table S1. Oligonucleotides.**

| Number | Name | Sequence 5’ - 3’ (restriction sites are underlined) | Description |
| --- | --- | --- | --- |
| ST16 | Seq pGFPamy II fwd | GGTAAGTTTTCCGTATGTTGC | Sequencing primer located in *gfpmut3* |
| ST17 | Seq pGFPamy II rev | GTGAATTTAGGAGGCTTACTTG | Sequencing primer located in integration vector backbone |
| ST39 | ycgB fwd | CTGGTCGGAGATTGGGATGATAG | Check for chromosomal *amyE* integration and sequencing |
| ST40 | ldh rev | AATTTCCATGTTGCGTAAGTCAG | Check for chromosomal *amyE* integration and sequencing |
| ST41 | cm fwd | ATGAACTTTAATAAAATTGATTTAG | Sequencing primer located in *cat* |
| ST129 | amp fwd | ATGAGTATTCAACATTTCCGTGTC | Check for single cross-over events via *bla* amplification |
| ST130 | amp rev | TTACCAATGCTTAATCAGTGAGG | Check for single cross-over events via *bla* amplification |
| ST135 | TgyrA fwd | TAACCGCGG*GGTACC*ATCGT*GGATCC*GTGTGAAAAAAAGCGCAGCTG | Amplification of T*gyrA*; SacII site; Small cloning site (SCS) including KpnI and BamHI sites in grey italics |
| ST147 | TgyrA II rev | AATCCGCGG*TAAA*ATGACTGTAAAGGGTTATGACAC | Amplification of T*gyrA;* SacII site; cured LIC site in grey italics |
| ST161 | pamy Gibson LIC fwd | GCTTTCCCGGGAAGGAGGAACTACTATG | Gibson primer for amplification of fluorophore containing part |
| ST162 | pamy Gibson LIC rev | CATAGTAGTTCCTCCTTCCCGGGAAAGC | Gibson primer for amplification of T*gyrA* containing part |
| ST163 | pamy Gibson Backbone fwd | GTACAATCTGCTCTGATGCCGCATAG | Gibson primer for amplification of T*gyrA* containing part |
| ST164 | pamy Gibson Backbone rev | CTATGCGGCATCAGAGCAGATTGTAC | Gibson primer for amplification of fluorophore containing part |
| ST165 | PrapE up fwd | *CCGCGGGCTTTCCCAGC*TAAATCCACCTCCGAAGG | Amplification of *rapE* promoter upstream part; LIC overlap in grey italics |
| ST166 | PrapE up rev | TAACTCTACTAGTACTGTAAATATTAACAAG | Amplification of *rapE* promoter upstream part |
| ST168 | PrapE down rev | *GTTCCTCCTTCCCACC*CCTCAATTTTATTTAAATATTCTG | Amplification of *rapE* promoter downstream part; LIC overlap in grey italics |
| ST169 | PrapE down fwd II | *TTAATATTTACAGTACTAGTAGAGTTA*ATATCAATCACATCAGCTGAAG | Amplification of *rapE* promoter downstream part; overlap for extension PCR in grey italics |
| ST189 | ldh Gibson mut del fwd | GGTATGTTTCTCTTTGATGTCTTTTTG | Deletion of *ldh* homologous sites via Gibson assembly |
| ST190 | ldh Gibson mut del rev | *AGACATCAAAGAGAAACATACC*GCGAGGCTGGATGGCCTTC | Deletion of *ldh* homologous sites via Gibson assembly; overlap for recircularization via Gibson assembly in grey italics |
| ST191 | Star Backbone Seq fwd | TTGAAGCTGTCCCTGATG | Sequencing primer for deletion of *ldh* homologous sites via Gibson assembly |
| ST192 | Star amyE Seq rev | CTTAAGCGGTTCTCTTCC | Sequencing primer for deletion of *ldh* homologous sites via Gibson assembly |
| ST193 | Star yfp Seq rev | GAACTTGTGGCCGTTTAC | Sequencing primer located in *iyfp* |
| ST194 | Star cfp Seq rev | TACGTAGCATCTCCTTCTCC | Sequencing primer located in *cfp_Bs_* |
| LA20 | CFP Seq | CAGTTCTTCGCCTTTTGAAAC | Sequencing primer located in *cfp_Bs_* |
| LA21 | IYFP Seq | TGAATCCATAGTAGTTCCTCC | Sequencing primer located in *iyfp* |
| LA38 | bglS up | CGGCTCTACAAAGACGAATTTG | Check for chromosomal *bglS* integration |
