# Supplementary material for: Ectopic Integration Vectors for Generating Fluorescent Promoter Fusions in Bacillus subtilis with Minimal Dark Noise
Source: PLoS One. 2014 May 29;9(5):e98360. doi: 10.1371/journal.pone.0098360 (PMC4038550; doi:10.1371/journal.pone.0098360)
Supplement: Table S2 — Enzymes and kits. (DOCX) [file pone.0098360.s002.docx]

**Supporting Information**

**Table S2. Enzymes and kits.**

| Enzymes / Kits | Company |
| --- | --- |
| Q5 DNA polymerase | New England Biolabs (Ipswich, MA, USA) |
| Phusion DNA polymerase | Thermo Scientific (Vilnius, Lithuania) |
| Taq DNA polymerase | Thermo Scientific (Vilnius, Lithuania) |
| Restriction enzymes | Fermentas/Thermo Scientific (Vilnius, Lithuania);  New England Biolabs (Ipswich, MA, USA) |
| DNA modifying enzymes | New England Biolabs (Ipswich, MA, USA) |
| QIAprep Spin Miniprep Kit for plasmid isolation and cleanup | Qiagen (Hilden, Germany) |
| QIAquick PCR Purification Kit | Qiagen (Hilden, Germany) |
| Wizard SV Gel and PCR Clean-Up System | Promega (Madison, WI, USA) |
| NucleoSpin Tissue Kit for genomic DNA isolation and cleanup | Macherey-Nagel (Düren, Germany) |
